# Baicalin alleviates lipopolysaccharide-induced neuroglial activation and inflammatory factors activation in hippocampus of adult mice

**DOI:** 10.1186/s42826-020-00058-w

**Published:** 2020-09-17

**Authors:** Murad-Ali Shah, Dong-Ju Park, Ju-Bin Kang, Myeong-Ok Kim, Phil-Ok Koh

**Affiliations:** 1 Department of Anatomy, College of Veterinary Medicine, Research Institute of Life Science, Gyeongsang National University, 501 Jinjudaero, Jinju, 52828 South Korea; 2 Division of Life Science and Applied Life Science, College of Natural Sciences, Gyeongsang National University, 501 Jinjudaero, Jinju, 52828 South Korea

**Keywords:** Baicalin, Hippocampus, Lipopolysaccharide, Neuroinflammation

## Abstract

Baicalin is a natural flavonoid that exerts a variety of pharmaceutical effects such as anti-inflammatory and antioxidant. Lipopolysaccharide (LPS) is an endotoxin that releases inflammatory cytokines and induces inflammatory response. This study was investigated the anti-inflammatory mechanism of baicalin against LPS-induced inflammatory response in the hippocampus. Adult mice were randomly grouped into control, LPS-treated, and LPS and baicalin co-treated animals. LPS (250 μg/kg/day) and baicalin (10 mg/kg/day) were administered intraperitoneally for 7 consecutive days. We measured neuroglia cells activation and inflammatory factors activation using Western blot analysis and immunofluorescence staining techniques. Ionized calcium binding adaptor molecule-1 (Iba-1) and glial fibrillary acidic protein (GFAP) are widely used as microglia and astrocyte markers, respectively. LPS treatment increased Iba-1 and GFAP expression, while baicalin co-treatment attenuated this overexpression. Nuclear factor-kappa B (NF-κB) is a key mediator of inflammation. Baicalin co-treatment alleviated LPS-induced increase of NF-κB in the hippocampus. In addition, LPS treatment upregulated pro-inflammatory cytokines including interleukin-1β (IL-1β) and tumor necrosis factor-α (TNF-α). However, baicalin co-treatment prevented LPS-induced increases of IL-1β and TNF-α in the hippocampus. Results from the present study showed that baicalin suppresses LPS-induced neuroinflammation by regulating microglia and astrocyte activation and modulating inflammatory factors in the hippocampus. Thus, these results demonstrate that baicalin has neuroprotective effect by alleviates microglia and astrocyte activation and modulates inflammatory response by suppressing NF-κB expression in hippocampus with neuroinflammation caused by LPS.

## Introduction

Inflammation is considered a defense response against infection or injury and an important feature for the treatment of infectious disease. Lipopolysaccharide (LPS) is a bacterial endotoxin that is found in gram-negative bacteria [1]. LPS activates neuroglial cells and induces the inflammatory response in the nervous system. Administration of LPS causes inflammation in brain tissue and subsequently leads to neuronal dysfunction [2]. Inflammation with bacterial infection leads to neurological diseases such as Parkinson’s disease [3]. In addition, LPS induces inflammation in many tissues including liver and lung [4, 5]. Inflammation is regulated by several inflammatory mediators such as cytokines and chemokines [6, 7]. LPS induces pro-inflammatory cytokines and leads to inflammatory response and cell death [8]. Pro-inflammatory cytokines act as critical factors in neuroinflammation and neurodegeneration processes of neurodegenerative diseases [9, 10].

Baicalin is a flavonoid compound derived from the roots of *Scutellaria baicalensis Georgi* and exerts a variety of beneficial effects in the central nervous system and immune system [11]. Baicalin has antioxidant, anti-inflammatory, anti-cancer, as well as anti-apoptotic properties and exerts neuroprotective effects on global cerebral ischemia and reperfusion models [12–14]. Baicalin improves the spatial learning ability by reducing hippocampal apoptosis. In addition, baicalin exerts anti-inflammatory effects by inhibiting the production of inflammatory mediators and binding to chemokines [13]. It exhibits anti-inflammatory properties against LPS-induced inflammation by suppressing the nuclear factor-kappa B (NF-κB) signaling pathway in HBE16 airway epithelial cells [15]. Moreover, baicalin prevents superantigen-induced inflammatory cytokines and chemokines [16]. Previous studies have been shown the anti-inflammatory and anti-apoptotic effects of baicalin in liver, lung, and kidney injuries [17, 18]. However, little information is demonstrated the anti-inflammatory effects of baicalin against LPS-induced toxicity in the hippocampus. The present study elucidates how baicalin regulates the inflammatory response caused by LPS in hippocampus of adult mice. We examined neuroglia cells activation during LPS-induced damage. This study was also investigated the anti-inflammatory mechanism of baicalin on LPS-induced expressions of NF-κB and pro-inflammatory factors, such as interleukin-1β (IL-1β) and tumor necrosis factor-α (TNF-α), in the hippocampus.

## Materials and methods

### Experimental animals and drug administration

Adult male mice (C57BL/6 N, 8 weeks, 31–33 g, *n* = 30) were obtained from Samtako Co. (Animal Breeding Center, Osan, Korea). All experimental procedures were carried out according to the guideline of the Institutional Animal Care and Use Committee of Gyeongsang National University. Mice were housed under controlled temperature (23–25 °C) and lighting condition (12 h light/ 12 h dark cycle) with free acess to food and water. Animals were divided into the following three groups: control group, LPS-treated group, LPS and baicalin co-treated group. LPS (250 μg/kg, Sigma Aldrich, St. Louis, MO, USA) was dissolved in normal saline solution and treated by intraperitoneal injection for 7 days. Baicalin (10 mg/kg, Sigma Aldrich) was dissolved in 0.1% dimethyl sulfoxide with normal saline and co-treated with LPS for 7 days. Vehicle was prepared with same solvent solution without baicalin and treated for consecutive 7 days in control group. Body weight was measured and animals were sacrificed at 24 h after last injection. Animals were euthanized by cervical dislocation and brain tissues were removed from skull and fixed in 4% neutral buffered paraformaldehyde (NBP) for immunofluorescence staining (*n* = 5). For Western blot analysis, hippocampal tissues were separated from brain and kept in − 70 °C (*n* = 5).

### Western blot analysis

Hippocampal tissues were homogenized and sonicated in lysis buffer [1% Triton X-100, 1 mM EDTA in phosphate-buffered saline (PBS) (pH 7.4)]. Homogenates were centrifuged at 15,000 g for 20 min and supernatants were collected. Protein concentration was determined by BCA protein analysis kit (Thermo Fischer Scientific, Walthem, MA, USA)). Protein samples (30 μg) were separated by 10% sodium dodecyl sulfate poly-acrylamide gels and transferred to a poly-vinylidene fluoride membrane (Millipore, Billerica, MA, USA). Membranes were incubated with 5% skim milk solution for 1 h and washed with tris-buffered saline containing 0.1% Tween-20 (TBST). They were incubated for overnight at 4 °C with following primary antibody: mouse anti-ionized calcium binding adaptor molecule-1 (Iba-1), mouse anti-glial fibrillary acidic protein (GFAP), mouse anti-NF-κB p65, mouse anti-TNF-α, mouse anti- IL-1β, and mouse anti- β-actin (1:1000, Santa Cruz Biotechnology, Dallas, TX, USA). Membranes were washed with TBST and reacted with horseradish peroxidase-conjugated anti-mouse IgG or anti-rabbit IgG (1:5000, Thermo Fischer Scientific) for 2 h at room temperature. They were washed with TBST and incubated with enhanced chemiluminescence reagent (GE Healthcare, Chicago, IL, USA). Immunoreactive protein bands were visualized on X-ray film and analyzed by a Image J (National Institutes of Health, Bethesda, MD, USA).

### Immunofluorescence staining

Fixed brain tissues with 4% NBP were washed with tap water for overnight. Brain tissues were dehydrated with a graded ethanol series (70 to 100%) and cleaned with xylene. They were embedded with paraffin in embedding center (Leica, Wetzlar, Germany) and blocked with paraffin according to a routine protocol. They were sliced into 4 μm thickness coronal section using a rotatory microtome (Leica). Sliced brain tissues were placed on slide glass, deparaffinized with xylene, rehydrated with a graded ethanol series (100 to 70%), and washed with PBS. They were incubated with normal goat serum for 1 h to block non-specific binding and reacted for overnight at 4 °C with following primary antibody: mouse anti-Iba-1, mouse anti-GFAP, mouse anti-NF-κB p65, mouse anti-TNF-α, and mouse anti-IL-1β (1:100, Santa Cruz Biotechnology). Slides were washed with PBS and incubated with fluorescein isothiocyanate (FITC)-conjugated anti-mouse IgG (1:200, Santa Cruz Biotechnology) for 2 h at room temperature. They were washed with PBS and reacted with 4′, 6-Diamidino-2-phenylindole (DAPI, Sigma Aldrich) for 10 min to counterstain nucleus. Slides were mounted with Ultra-Cruz mounting medium (Santa Cruz Biotechnology). Positive signals were observed using a confocal microscope (Flouview FV 1000, Olympus) and photographed. The number of FITC and DAPI double positive cells in hippocampal CA1, CA2 and CA3 areas were counted by blinded experimenter. Counted cell numbers were calculated and expressed as average number of FITC and DAPI double positive cells in each hippocampal area.

### Statistical analysis

All data from the experiment are expressed as mean ± standard error of mean (S.E.M.). The experiment results in each group were compared by two-way analysis of variance (ANOVA) followed by post-hoc Scheffe’s test. A value of *p* < 0.05 was regarded as statistically significant.

## Results

**Fig. 1:**
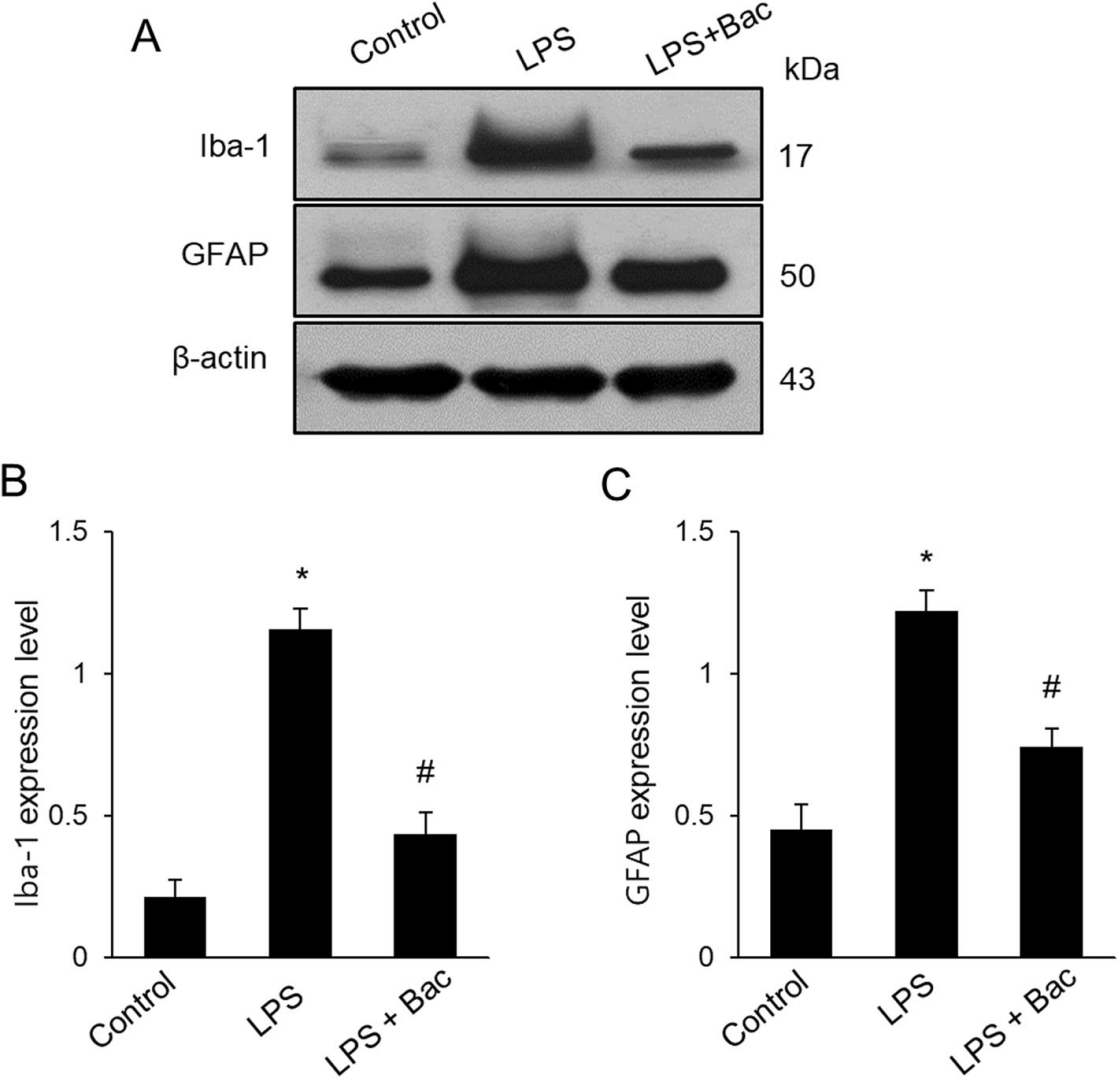
Western blot analysis of ionized calcium binding adaptor molecule-1 (Iba-1) and glial fibrillary acidic protein (GFAP) in hippocampus of control, lipopolysaccharide (LPS)-treated, and LPS and baicalin (Bac) co-treated animals (**a**). Density values of Western blot analysis are expressed as a ratio of these proteins intensities to β-actin intensity (**b**, **c**). Data (*n* = 5) are shown as mean ± S.E.M. * *p* < 0.05 vs. control animal, # *p* < 0.05 vs. LPS-treated animal

**Fig. 2:**
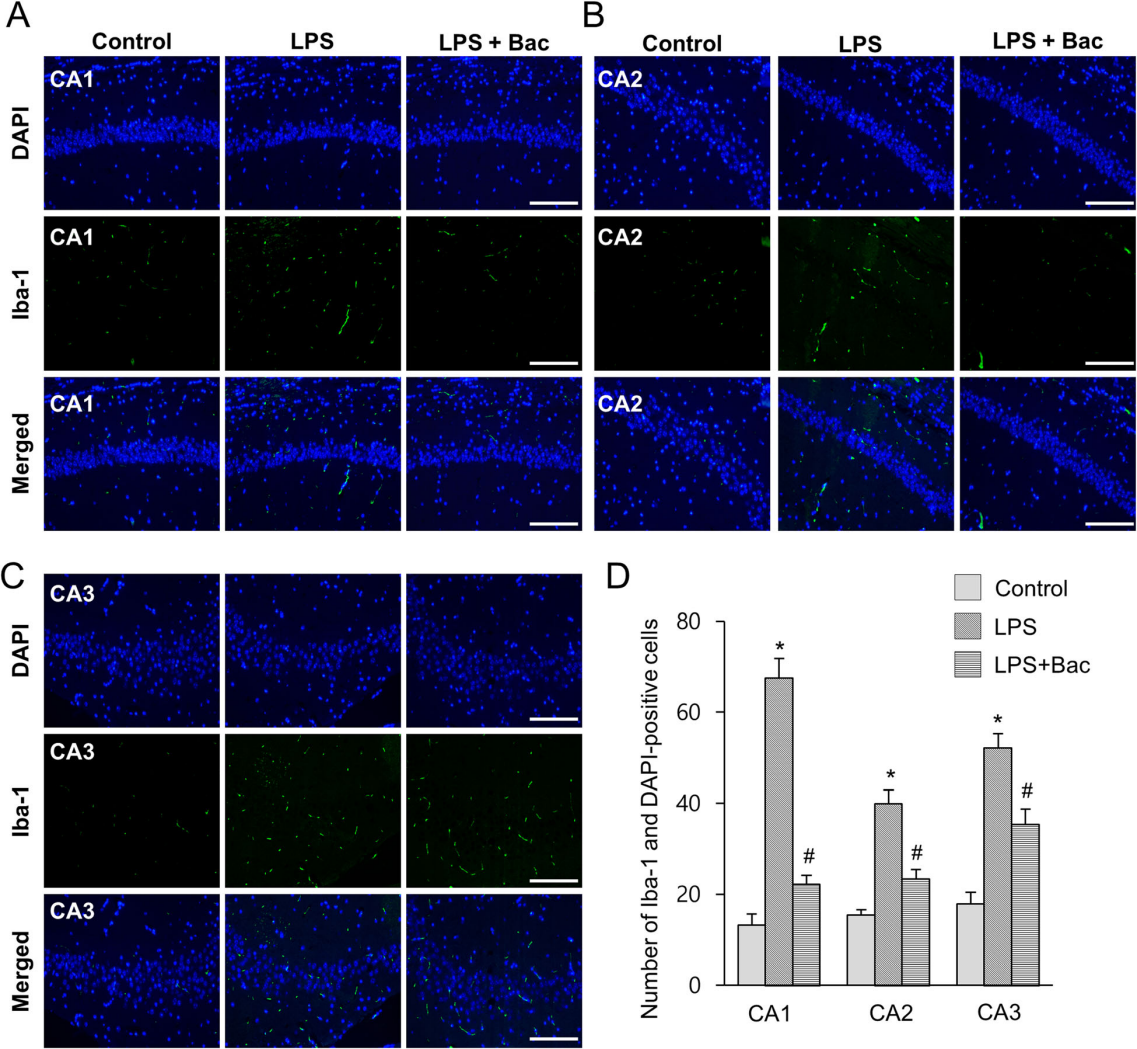
Double immunofluorescence labeling of ionized calcium binding adaptor molecule-1 (Iba-1) in hippocampus of control, lipopolysaccharide (LPS)-treated, and LPS and baicalin (Bac) co-treated animals (**a**-**c**). Scale bar = 100 μm. Iba-1 and DAPI-positive cells in hippocampal CA1, CA2 and CA3 region (**d**). Data (*n* = 5) are shown as mean ± S.E.M. * *p* < 0.05 vs. control animal, # *p* < 0.05 vs. LPS-treated animal

**Fig. 3:**
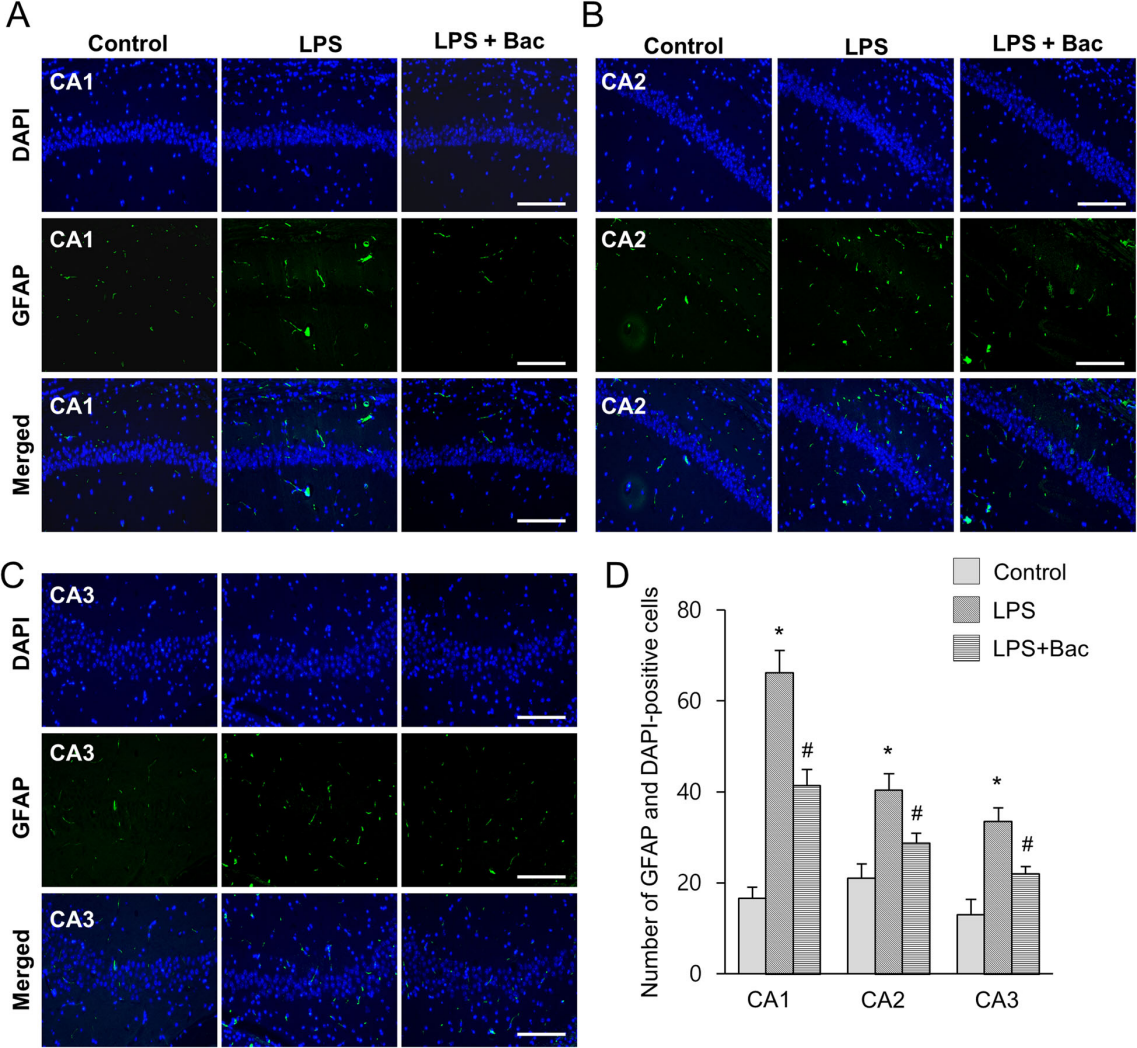
Double immunofluorescence labeling of glial fibrillary acidic protein (GFAP) in hippocampus of control, lipopolysaccharide (LPS)-treated, and LPS and baicalin (Bac) co-treated animals (**a**-**c**). Scale bar = 100 μm. GFAP and DAPI-positive cells in hippocampal CA1, CA2 and CA3 region (**d**). Data (*n* = 5) are shown as mean ± S.E.M. * *p* < 0.05 vs. control animal, # *p* < 0.05 vs. LPS-treated animal

We measured body weight as a basic physiological data. LPS reduced body weight, but baicalin attenuated weight loss induced by LPS. Body weights of mice were 31.4 ± 1.53 g in the LPS-treated and 35.8 ± 1.45 g in baicalin co-treated animals after 7 days injection. Moreover, body weights of the control group were 37.8 ± 2.45 g. Iba-1 and GFAP expression was investigated to confirm the activation of microglia and astrocytes in the hippocampus. Increases in Iba-1 and GFAP expression were found in the hippocampus of LPS-treated animals. However, LPS and baicalin co-treatment significantly attenuated LPS-induced increases in Iba-1 and GFAP expression (Fig. 1a). Western blot analysis showed that Iba-1 expression levels were 1.15 ± 0.07 and 0.43 ± 0.06 in LPS-treated and baicalin co-treated animals, respectively (Fig. 1b). In addition, GFAP expression levels were 1.21 ± 0.07 in LPS-treated and 0.74 ± 0.08 in baicalin co-treated animals (Fig. 1c). Results from immunofluorescence staining showed increased positive reactions of Iba-1 and GFAP in LPS-treated animals; the increases were significantly attenuated in LPS and baicalin co-treated animals (Figs. 2, 3). These changes appeared in every region including CA1, CA2, and CA3. These changes in the CA1 region are more severe than CA2 and CA3 regions. In the CA1 region, the number of Iba-1 and DAPI double positive cells were 67.6 ± 4.28 and 22.2 ± 1.92 in LPS-treated and baicalin co-treated animals, respectively (Fig. 2d). Moreover, the number of GFAP and DAPI double positive cells were 66.2 ± 4.92 in LPS-treated and 41.4 ± 3.51 in baicalin co-treated animals (Fig. 3d).

**Fig. 4:**
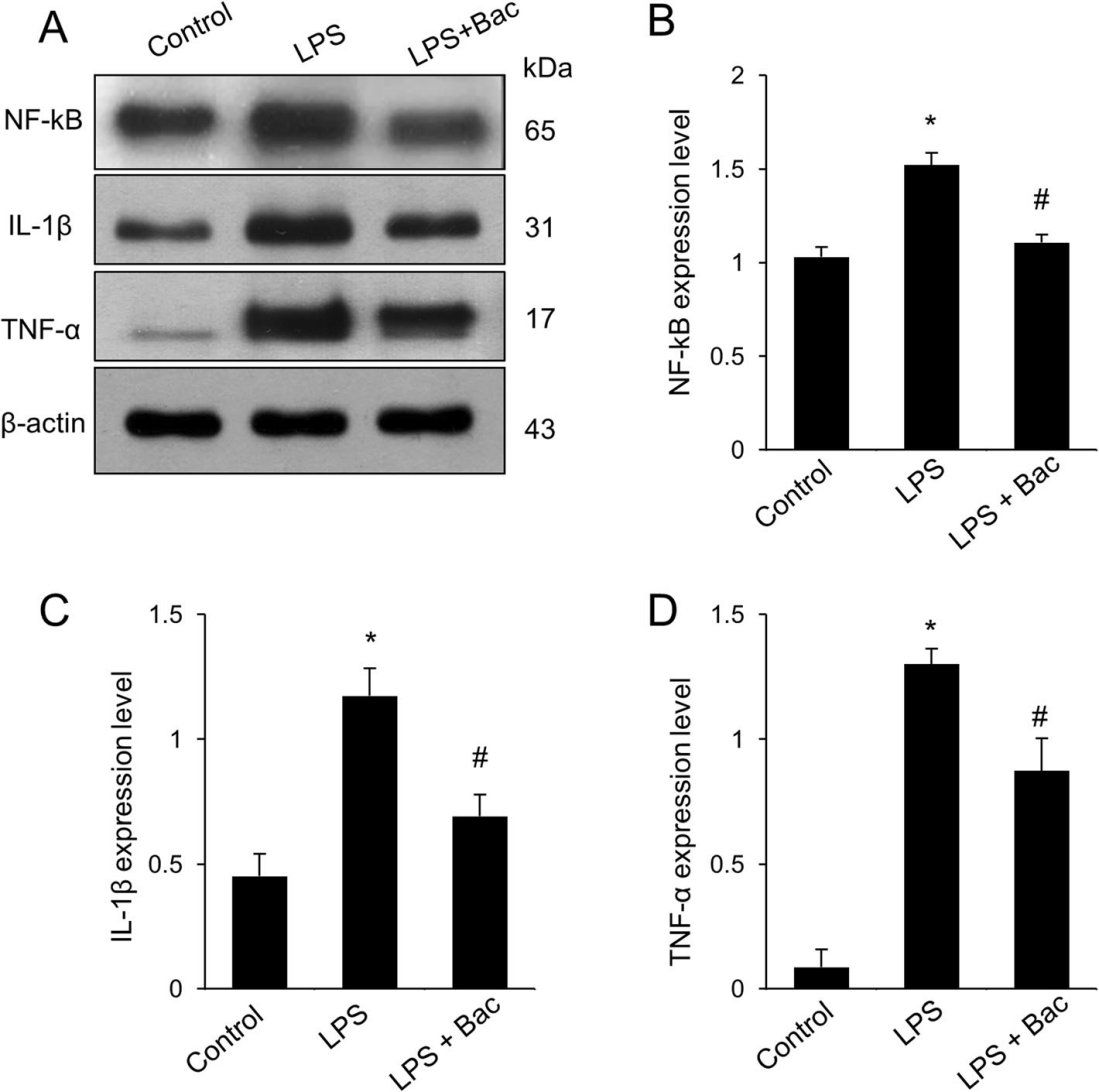
Western blot analysis of nuclear factor kappa B (NF-κB), interleukin-1β (IL-1β), and tumor necrosis factor-α (TNF-α) in hippocampus of control, lipopolysaccharide (LPS)-treated, and LPS and baicalin (Bac) co-treated animals (**a**). Density values of Western blot analysis are expressed as a ratio of these proteins intensities to β-actin intensity (**b**-**d**). Data (*n* = 5) are shown as mean ± S.E.M. * *p* < 0.05 vs. control animal, # *p* < 0.05 vs. LPS-treated animal

**Fig. 5:**
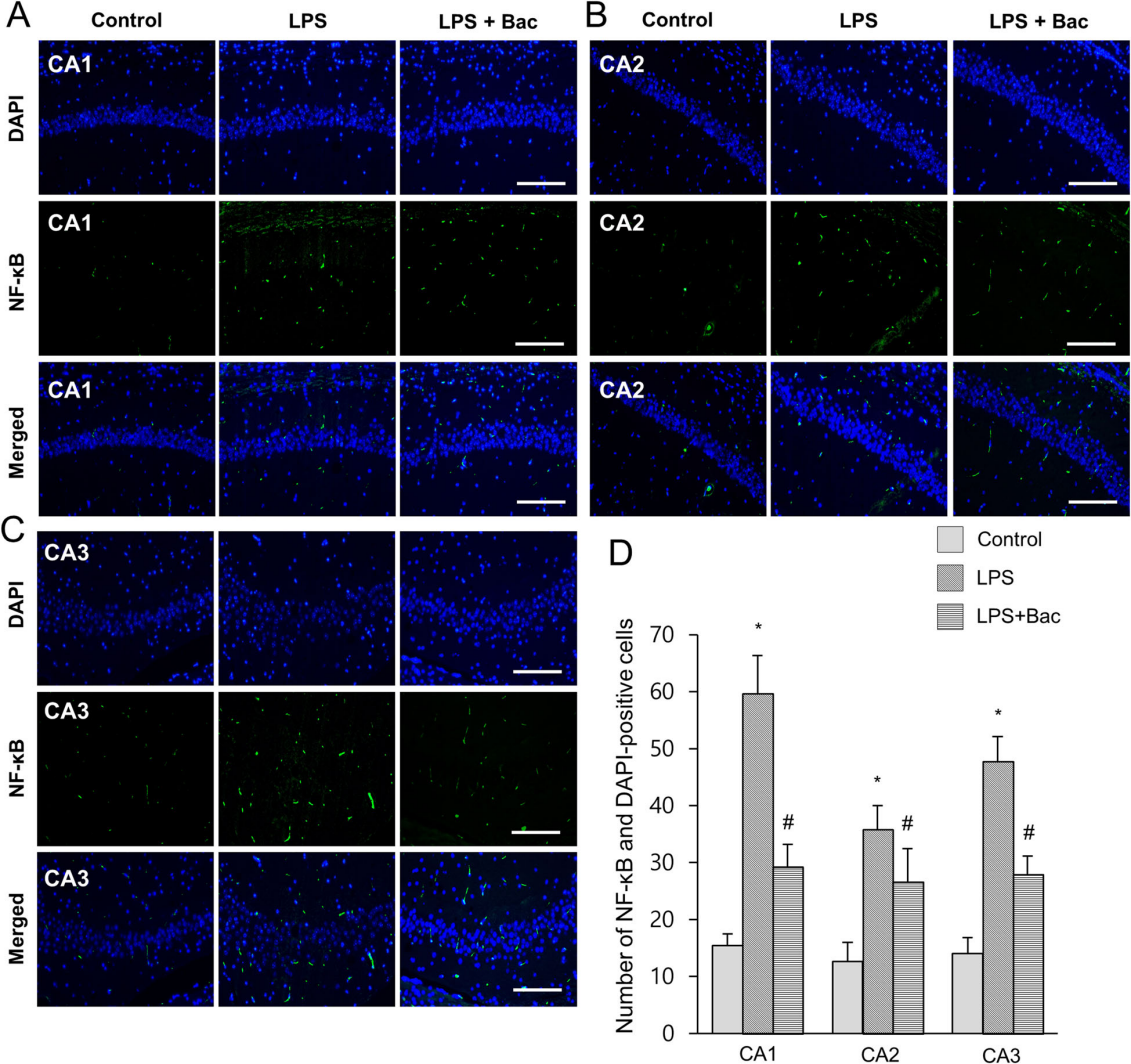
Double immunofluorescence labeling of nuclear factor kappa B (NF-κB) in hippocampus of control, lipopolysaccharide (LPS)-treated, and LPS and baicalin (Bac) co-treated animals (**a**-**c**). NF-κB and DAPI-positive cells in hippocampal CA1, CA2 and CA3 region (**d**). Data (*n* = 5) are shown as mean ± S.E.M. Scale bar = 100 μm. * *p* < 0.05 vs. control animal, # *p* < 0.05 vs. LPS-treated animal

**Fig. 6:**
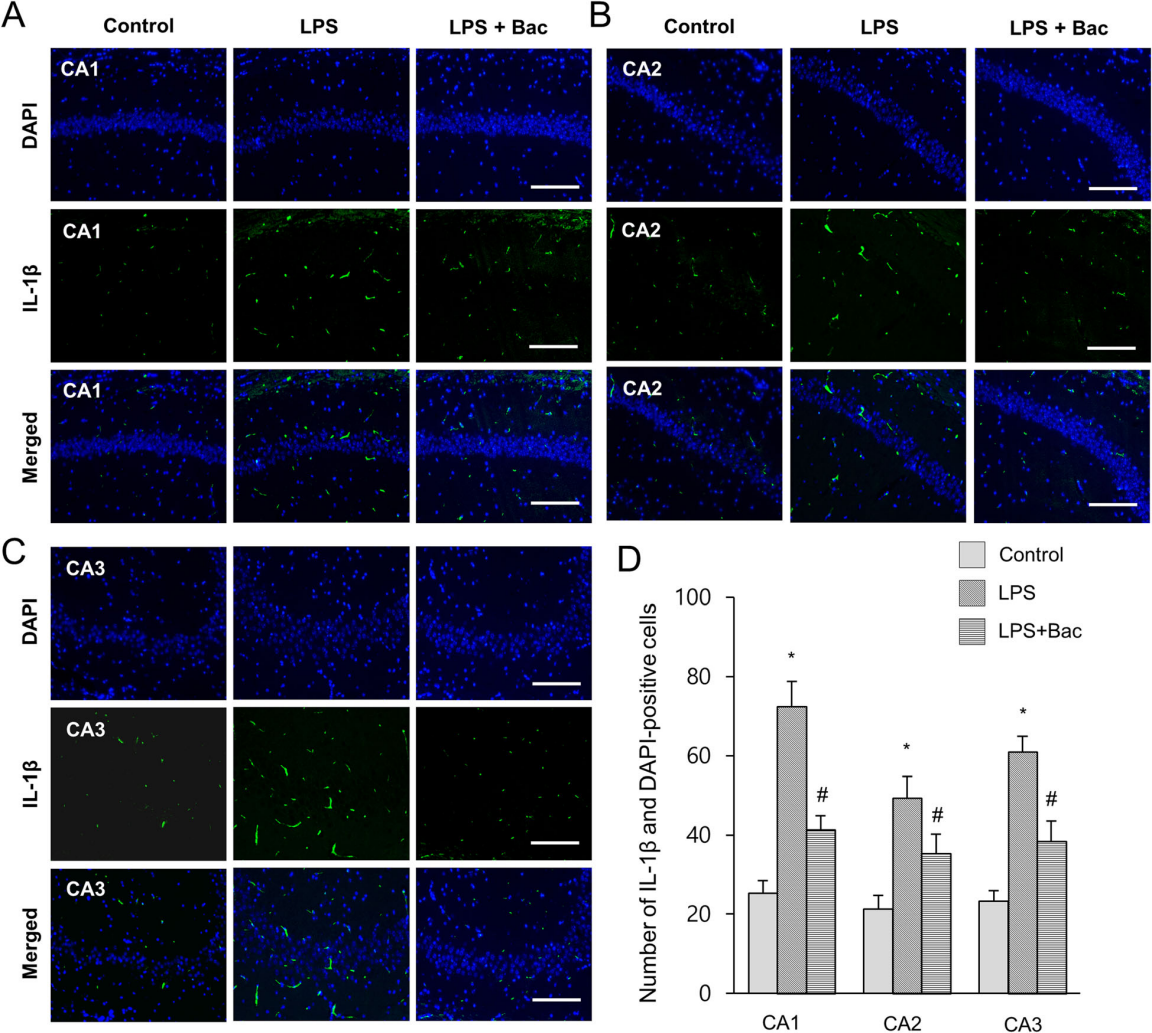
Double immunofluorescence labeling of interleukin-1β (IL-1β) in hippocampus of control, lipopolysaccharide (LPS)-treated, and LPS and baicalin (Bac) co-treated animals (**a**-**c**). IL-1β and DAPI-positive cells in hippocampal CA1, CA2 and CA3 region (**d**). Data (*n* = 5) are shown as mean ± S.E.M. Scale bar = 100 μm. * *p* < 0.05 vs. control animal, # *p* < 0.05 vs. LPS-treated animal

**Fig. 7:**
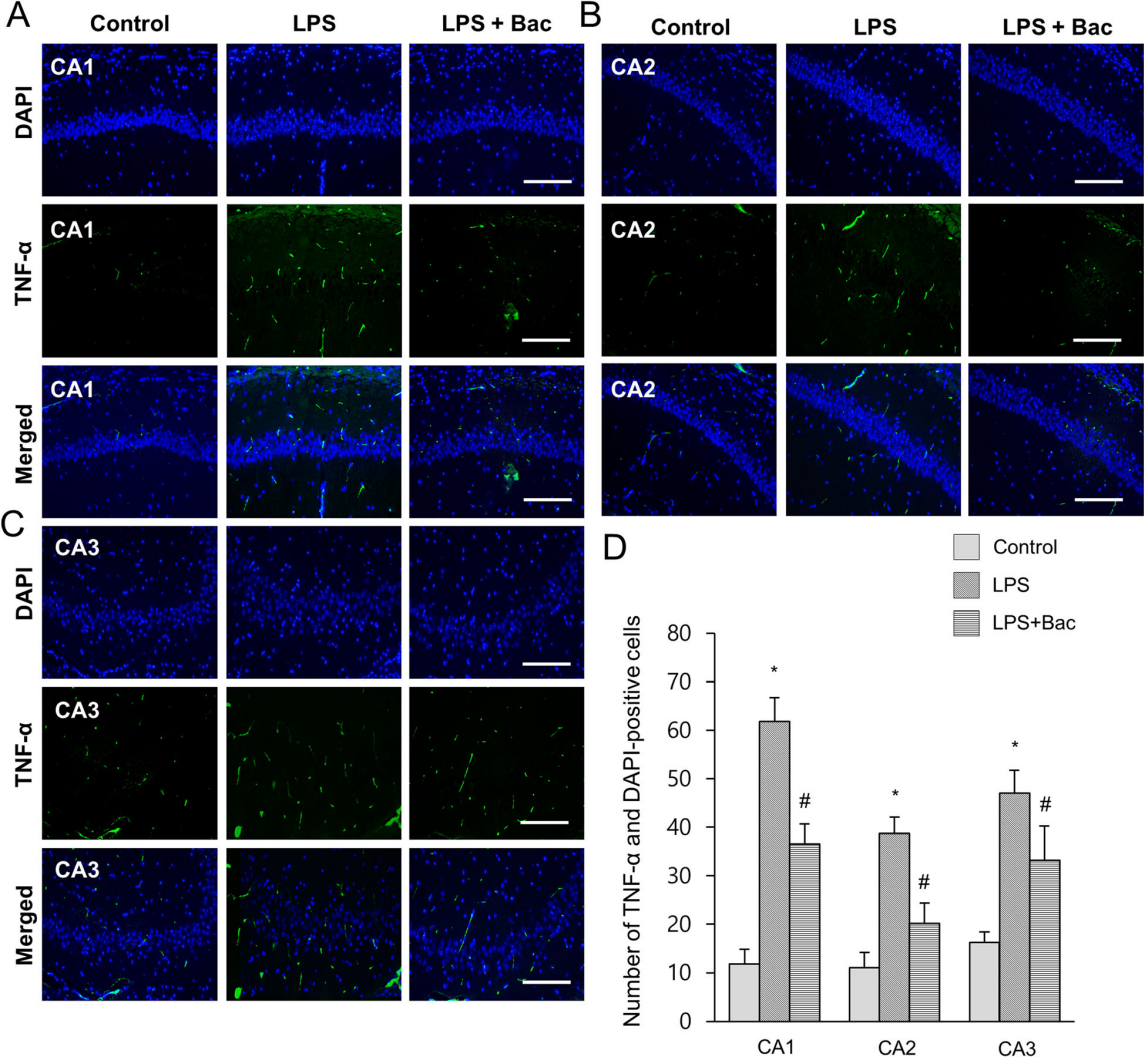
Double immunofluorescence labeling of tumor necrosis factor-α (TNF-α) in hippocampus of control, lipopolysaccharide (LPS)-treated, and LPS and baicalin (Bac) co-treated animals (**a**-**c**). TNF-α and DAPI-positive cells in hippocampal CA1, CA2 and CA3 region (**d**). Data (*n* = 5) are shown as mean ± S.E.M. Scale bar = 100 μm. * *p* < 0.05 vs. control animal, # *p* < 0.05 vs. LPS-treated animal

Figure 4a showed the changes of NF-κB in LPS and baicalin co-treated animals. Western blot analysis showed that NF-κB expression levels were 1.52 ± 0.07 and 1.11 ± 0.04 in LPS-treated and baicalin co-treated animals, respectively (Fig. 4b). Furthermore, changes of inflammatory cytokines, including IL-1β and TNF-α, were observed in LPS and baicalin co-treated animals (Fig. 4a). Results from immunoblotting showed increased IL-1β and TNF-α protein expression levels in the hippocampus of LPS-treated animals. However, LPS and baicalin co-treatment significantly attenuated the increased IL-1β and TNF-α inflammatory cytokine expression levels. IL-1β expression levels were 1.17 ± 0.11 in LPS-treated and 0.69 ± 0.08 in baicalin co-treated animals (Fig. 4c). TNF-α expression levels were 1.30 ± 0.06 and 0.87 ± 0.13 in LPS-treated and baicalin co-treated animals, respectively (Fig. 4d). Changes in NF-κB, IL-1β, and TNF-α levels were confirmed based on immunofluorescence staining results (Figs. 5-7). These results showed a pattern similar to immunoblotting data. These changes were observed in CA1, CA2, and CA3 regions. NF-κB, IL-1β, and TNF-α expressions were more significantly increased in the CA1 region than CA2 and CA3 regions. In the CA1 region, the numbers of NF-κB and DAPI double positive cells were 59.6 ± 6.77 and 29.2 ± 3.96 in LPS-treated and baicalin co-treated animals, respectively (Fig. 5d). The numbers of IL-1β and DAPI double positive cells were 72.4 ± 6.35 in LPS-treated and 41.4 ± 3.51 in baicalin co-treated animals (Fig. 6d). The numbers of TNF-α and DAPI double positive cells were 61.8 ± 4.92 and 36.6 ± 4.04 in LPS-treated and baicalin co-treated animals, respectively (Fig. 7d).

## Discussion

Baicalin has anti-inflammatory effects against various inflammatory diseases [13]. Our results confirmed the anti-inflammatory property of baicalin against LPS-treatment in hippocampal tissues of adult mice. Systemic administration of LPS leads to activation of microglia and astrocytes [19, 20]. Activated microglia causes the production of pro-inflammatory cytokines leading to neuroinflammation and neurodegeneration [10]. Baicalin exerts an anti-inflammatory effect by suppression of inflammasome activation in LPS-exposed piglet mononuclear phagocytes [21]. LPS activates generation of pro-inflammatory cytokines and induces neuroinflammation and neurodegeneration [22]. LPS leads to activation of microglia and astrocytes in the hippocampus. Iba-1 and GFAP are used as markers of activated microglia and astrocytes [23]. Moreover, baicalin attenuated LPS-induced increases in Iba-1 and GFAP expression in the hippocampus. Our results indicated that baicalin alleviates LPS-induced activation of microglia and astrocytes in the hippocampus. Iba-1 acts as an adaptor molecule facilitating calcium signals and abundantly exists in microglia and macrophages [24]. It is significantly upregulated in activated microglial cells [25]. In addition, GFAP is a filamentous protein in astrocytes and involved in the structure and function of the cell cytoskeleton. GFAP contributes to the mechanical strength and shape of cells, formation of the blood-brain barrier, and communication of cells. GFAP-knockout mice displayed abnormal myelination and impaired blood-brain barrier [26]. Baicalin alleviated LPS-induced increase in GFAP expression. Our results from the present study showed that baicalin prevents increased Iba-1 and GFAP expression levels caused by LPS in the hippocampus, suppresses activation of microglia and astrocytes, and modulates neuroinflammation.

Inflammation is a protective immune mechanism against infection caused by foreign organisms, such as bacteria and viruses. However, excessive inflammatory response leads to systemic dysfunction and tissue damage, finally causing cell death. Macrophages have an important role in initiation and maintenance of inflammation and production of pro-inflammatory cytokines [27, 28]. Pro-inflammatory cytokines, such as TNF-α and IL-1β, are critical factors in pathogenesis of the inflammatory response. In addition, the inflammatory response is a pathological process which can cause many inflammatory diseases and regulated by the NF-κB signaling pathway [29]. NF-κB is a protein complex that regulates DNA transcription, cytokine production, and cell survival. NF-κB plays a critical role in modulating the immune response to infection. Abnormal modulation of NF-κB is associated with inflammatory and autoimmune diseases, viral infection, and improper immune development. NF-κB is activated by exposure of various inflammatory cytokines such as TNF-α, IL-1β, and oxidative stress [30, 31]. LPS leads to the activation of pro-inflammatory mediators and causes neuroinflammation [32]. In the present study, LPS contributed to upregulation of NF-κB in the hippocampus and baicalin co-treatment mitigated the increase of NF-κB. These results showed that baicalin attenuates the LPS-induced neuroinflammation by regulating NF-κB expression.

IL-1β belongs to the interleukin-1 family and is a major mediator of inflammation [33]. IL-1β is synthesized in various cells including monocytes, macrophages, and neutrophils. IL-1β binds to interleukin 1 receptor, initiates a cascade of inflammatory cytokines and chemokines, and results in the inflammatory response [34]. LPS increases the expression of IL-1β in bronchial epithelial cells and umbilical vein endothelial cells [35]. Our results showed LPS induces IL-1β production in the hippocampus. LPS treatment is associated with memory deficit, anxiety, depression, and neuroinflammation [36]. IL-1β acts as a key mediator in LPS-induced dysfunction of hippocampal neurons [37]. IL-1β knockdown significantly attenuated the memory deficits and anxiety- and depression-like behaviors caused by LPS. In addition, IL-1β knockdown ameliorated LPS-induced neuroinflammatory responses and abolished the downregulation of brain-derived neurotrophic factor [38, 39]. Our results showed that upregulation of IL-1β expression by LPS administration was attenuated by baicalin co-treatment in hippocampus. Baicalin mitigated neuroinflammation in the hippocampus caused by LPS treatment by regulating IL-1β expression. TNF-α is a potent inflammatory mediator that has an essential role in the inflammatory system. TNF-α is responsible for cytokine production, adhesion molecule expression, and growth stimulation [40]. TNF-α expression is upregulated in arthritis caused by LPS treatment [41]. Furthermore, LPS increased TNF-α levels in mouse neuronal cells [42]. Our results were in agreement with previous studies. Increased TNF-α expression was observed in the LPS-treated hippocampus and alleviated with baicalin co-treatment. Baicalin mitigated LPS-induced upregulation of NF-κB expression and inflammatory cytokines including IL-1β and TNF-α. In addition, baicalin suppressed the inflammatory response by preventing overactivation of microglia and astrocytes, regulating NF-κB expression and the inflammatory cytokines, IL-1β and TNF-α.

Hippocampal CA1 region is a sensitive area that responds to LPS stimuli [43]. Administration of LPS induces changes in synaptic transmission and plasticity in the hippocampal CA1 region [44]. In addition, superoxide induces selective vulnerability of the hippocampal CA1 region to damage, a more vulnerable area than CA3 region [45]. Immunofluorescence staining showed changes in Iba-1, GFAP, NF-κB, IL-1β, and TNF-α expression were more significantly increased in the CA1 region of LPS-treated animals than in the CA2 and CA3 regions. This study elucidated that baicalin attenuates LPS-induced inflammatory response by modulating microglia and astrocyte activation and inhibiting NF-κB expression and suppressing IL-1β and TNF-α expression in hippocampus.

## Conclusions

Our results showed that baicalin alleviates LPS-induced inflammatory response in the hippocampus by suppressing NF-κB associated with the inflammatory response. These results provide an evidence that baicalin modulates LPS-induced neuroinflammation in hippocampus of adult mice. Our finding also suggest the fact that baicalin has a potential therapeutic application for treating neuroinflammation.

## Data Availability

The data that support the findings of this study are available on request from the corresponding author on reasonable request.

## Abbreviations

DAPI: 4′, 6-Diamidino-2-phenylindole
FITC: Fluorescein isothiocyanate
GFAP: Glial fibrillary acidic protein
Iba-1: Ionized calcium binding adaptor molecule-1
IL-1β: Interleukin-1β
LPS: Lipopolysaccharide
NBP: Neutral buffered paraformaldehyde
NF-κB: Nuclear factor-kappa B
PBS: Phosphate-buffered saline
TBST: Tris-buffered saline containing 0.1% Tween-20
TNF-α: Tumor necrosis factor-α
